# Photosynthetic Acclimation to Fluctuating Irradiance in Plants

**DOI:** 10.3389/fpls.2020.00268

**Published:** 2020-03-24

**Authors:** Alejandro Morales, Elias Kaiser

**Affiliations:** ^1^Centre for Crop Systems Analysis, Plant Science Group, Wageningen University and Research, Wageningen, Netherlands; ^2^Plant Ecophysiology, Institute of Environmental Biology, Utrecht University, Utrecht, Netherlands; ^3^Molecular Plant Physiology, Institute of Environmental Biology, Utrecht University, Utrecht, Netherlands; ^4^Horticulture and Product Physiology, Plant Science Group, Wageningen University and Research, Wageningen, Netherlands

**Keywords:** fluctuating light, acclimation, dynamic photosynthesis, gene transcription, signaling

## Abstract

Unlike the short-term responses of photosynthesis to fluctuating irradiance, the long-term response (i.e., acclimation) at the chloroplast, leaf, and plant level has received less attention so far. The ability of plants to acclimate to irradiance fluctuations and the speed at which this acclimation occurs are potential limitations to plant growth under field conditions, and therefore this process deserves closer study. In the first section of this review, we look at the sources of natural irradiance fluctuations, their effects on short-term photosynthesis, and the interaction of these effects with circadian rhythms. This is followed by an overview of the mechanisms that are involved in acclimation to fluctuating (or changes of) irradiance. We highlight the chain of events leading to acclimation: retrograde signaling, systemic acquired acclimation (SAA), gene transcription, and changes in protein abundance. We also review how fluctuating irradiance is applied in experiments and highlight the fact that they are significantly slower than natural fluctuations in the field, although the technology to achieve realistic fluctuations exists. Finally, we review published data on the effects of growing plants under fluctuating irradiance on different plant traits, across studies, spatial scales, and species. We show that, when plants are grown under fluctuating irradiance, the chlorophyll a/b ratio and plant biomass decrease, specific leaf area increases, and photosynthetic capacity as well as root/shoot ratio are, on average, unaffected.

## Introduction

Fluctuations in irradiance are ubiquitous in nature, and they impact photosynthesis, water use, and plant growth. Ever since the realization that short-term (seconds-minutes) responses of photosynthesis to these fluctuations were under genetic control (Cruz et al., 2016) and that the speed of these responses could be improved by exploiting natural genetic variation (Qu et al., 2016; Soleh et al., 2017; Salter et al., 2019) or gene editing techniques to increase growth in the field (Kromdijk et al., 2016), research interest in this topic has been immense, as exemplified by the reviews published on it in recent years (Lawson and Blatt, 2014; Kaiser et al., 2015, 2018b, 2019; Armbruster et al., 2017; Slattery et al., 2018; Burgess et al., 2019; Simkin et al., 2019; Tanaka et al., 2019). Given this interest, it is surprising that the long-term response to irradiance fluctuations, i.e., photosynthetic acclimation (in the scale of days), has received relatively limited attention. For example, to our knowledge this is the first review to emphasize the long-term acclimation of plants to fluctuating irradiance. Importantly, enhancing the capacity to optimally acclimate to irradiance fluctuations and the speed at which acclimation happens could be another approach to improving plant growth in the field.

The most extensive work on acclimation to fluctuating irradiance was recently performed by Lawson and co-workers, who found that *Arabidopsis thaliana* plants acclimated to fluctuating irradiance showed reductions in biomass, leaf thickness, photosynthetic capacity, and concentrations of thylakoid proteins (Vialet-Chabrand et al., 2017) as well as changes in stomatal kinetics (Matthews et al., 2018). While these results highlight the importance of photosynthetic acclimation to irradiance fluctuations for photosynthesis and the plant, these responses may vary across species, frequency of fluctuation, and other environmental factors as yet unidentified, for which a broader analysis is needed.

In this review, we summarize the state-of-the-art on (i) the causes and characteristics of natural irradiance fluctuations which should be used to design better fluctuating irradiance protocols in the lab, (ii) the mechanisms of acclimation from which genetic improvements may be attempted, and (iii) an overview of studies published so far on long-term effects of fluctuating irradiance on plant growth: we review the methodology used in these studies (with emphasis on how fluctuations in irradiance were achieved) and provide an overview of effects of fluctuating irradiance on different plant traits, across studies, spatial scales, and species.

## Natural Irradiance Fluctuations and Circadian Rhythms Affect Dynamic Photosynthesis

### Changes in Natural Irradiance

**FIGURE 1:**
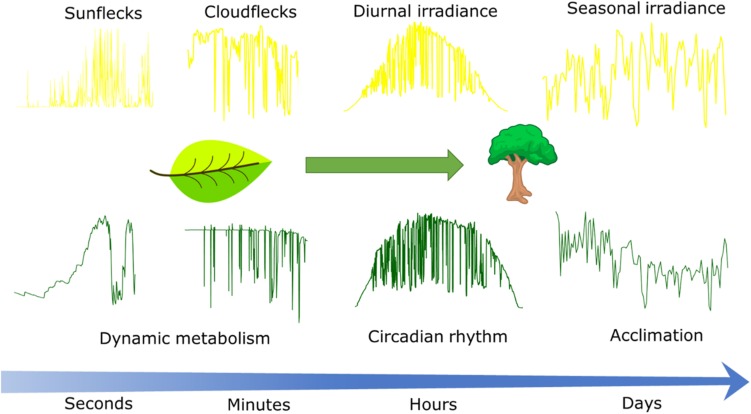
Conceptual scheme describing the time scales at which irradiance fluctuates in the field **(Upper)** and responses of CO_2_ assimilation to these fluctuations **(Lower)**. Leaf CO_2_ assimilation in response to sunflecks and cloudflecks as driven by dynamic metabolism was simulated with the dynamic photosynthesis model by Morales et al. (2018a). Canopy CO_2_ assimilation in response to diurnal and seasonal weather was simulated with the sun-shade canopy photosynthesis model by De Pury and Farquhar (1997) and happens at the timescales at which circadian rhythms (hours) and the effects of acclimation (days) are relevant. Time series of irradiance from weather station in Wageningen, Netherlands, except for the sunflecks time series that was measured by the authors with a portable light sensor placed beneath a durum wheat canopy during a clear summer day in Wageningen.

Irradiance projected onto the Earth’s surface varies during the day and season (Figure 1) mainly due to changes in the solar incident angle and changes in atmospheric transmissivity due to clouds and aerosols (Wald, 2018). Seasonal variability in irradiance increases with latitude due to larger variations in daylength and average solar incident angle, but synoptic weather patterns also introduce significant variation in atmospheric transmissivity (Parding et al., 2016). Generally, the variability in irradiance decreases with the timescale of integration (Perez et al., 2016).

Accurate empirical and mechanistic models for diurnal and seasonal solar radiation under clear skies exist (Ruiz-Arias and Gueymard, 2018), but predicting the effect of clouds on irradiance fluctuations remains challenging. For a given location, these effects can follow statistical trends, allowing the use of predictive models at the seasonal (Kafka and Miller, 2019) and daily timescales (Wang et al., 2016). Additionally, recent modeling efforts allow for simulating dynamic diurnal cloud formation and effects of these clouds on the spatial and temporal distribution of surface irradiance (Sikma et al., 2018).

Much of the recent research on variability in irradiance is driven by the needs of the solar energy sector (Victoria and Andresen, 2019), and plant science could benefit from that knowledge. For example, in a partially cloudy sky, the movement of clouds can cause strong fluctuations superimposed on the diurnal temporal pattern (cloudflecks; Figure 1). Understanding the distribution of cloudfleck duration and irradiance reduction is important, as it affects the response of photosynthesis, even if the total irradiance remains the same (Kaiser et al., 2016; Morales et al., 2018a). However, to our knowledge, very few studies have quantified the characteristics of cloudflecks from the perspective of photosynthesis (Knapp and Smith, 1988; Kaiser et al., 2018b).

The fluctuations in irradiance will also depend on the spatial scale under consideration. Whereas the models and studies cited above focus on understanding irradiance on the surface of the Earth based on data from irradiance sensors, photosynthesis occurs in chloroplasts within the leaves. Gaps in the canopy that expose leaves to the sun and which depend on plant architecture and/or movements of plants by wind introduce additional fluctuations in the irradiance incident on that leaf (Kaiser et al., 2018b), known as sunflecks (Figure 1). Partial exposure to the sun may also result in fluctuations due to the penumbra effect (Smith et al., 1989). Additionally, simulations of irradiance distribution within the leaf using a ray-tracing approach suggest that fluctuations may be enhanced at the chloroplast level, due to the heterogeneity of the light environment within leaves (Xiao et al., 2016), but an experimental confirmation of such an enhancement is currently lacking.

The duration and amplitude of sunflecks depends on wind speed (Tang et al., 1988; Roden, 2003), canopy structure (Peressotti et al., 2001; Kaiser et al., 2018b), plant biomechanical properties (Burgess et al., 2016, 2019), and position within the canopy (Pearcy et al., 1990). These fluctuations may be simulated with detailed 3D reconstructions of canopies coupled with physically based ray tracing algorithms, but challenges remain in the realistic simulation and measurement of plant movements by wind (Burgess et al., 2016; Retkute et al., 2018; Gibbs et al., 2019). The statistical properties of sunflecks were reviewed by Kaiser et al. (2018b) showing that sunflecks are generally short (<2 s).

Depending on the source responsible for the fluctuation in irradiance, there will also be a fluctuation in the spectral composition of the irradiance. Small changes in the spectrum will occur in cloudflecks due to differences in spectra of clouds, sun, and sky, but larger changes are associated with sunflecks due to the optical properties of leaves (Endler, 1993). A leaf under green shade is exposed to a higher relative proportion of green and far red (>700 nm) irradiance compared to direct exposure to the sky or direct solar irradiance. Thus, the transition from low to high irradiance during a sunfleck will also result in a rapid change in the red:far red ratio (R/FR) and in the proportion of green irradiance.

### Short-Term Responses of Photosynthesis to Irradiance Changes

The dynamic response of leaf photosynthesis to sunflecks and cloudflecks (the “short-term response” of photosynthesis) is determined by the dynamic regulation of enzyme and electron transport activities, metabolite buffering, CO_2_ diffusion, light harvesting capacity, non-photochemical quenching, and chloroplast movements (Kaiser et al., 2015, 2018b, 2019). While these responses are well characterized at the leaf level for C3 species (Morales et al., 2018a, b), less is known about the responses in C4 and CAM plants (Kaiser et al., 2018b). The short-term response of photosynthesis to fluctuating irradiance is known to be modulated by air temperature, humidity, soil salinity, CO_2_ concentration, and far red irradiance (Kono et al., 2019), but significant knowledge gaps remain (Kaiser et al., 2015; Zhang et al., 2018).

### Circadian Rhythms

Circadian rhythms in photosynthesis (Hennessey et al., 1993; Dodd et al., 2004; Resco de Dios et al., 2016) may contribute 15–25% of the diurnal variation across species and environments (Resco de Dios and Gessler, 2018). Also, in the evening, stomata opened faster and closed more slowly in response to increases and decreases in irradiance, respectively, regardless of the light regime (constant, sinusoidal, or fluctuating) that the plants were grown under (Matthews et al., 2018). Circadian rhythms have also been observed at the levels of photosynthetic metabolites (Fredeen et al., 1991) and sugars (Graf et al., 2010), and circadian rhythms in photosynthetic products may be partially responsible for driving circadian rhythms in gene expression (Dodd et al., 2015; Haydon and Webb, 2016).

## Acclimation Under Fluctuating Irradiance: Signaling, Gene Expression, Protein Abundance, and Kinetics

In this review, we follow Smith and Dukes (2013) who define acclimation as “a physiological, structural, or biochemical adjustment by an individual plant in response to an environmental stimulus that is manifested as alterations in the short-term response function of a physiological process”. Therefore, we consider as part of acclimation of photosynthesis to fluctuating irradiance any reversible physiological process or irreversible developmental process that affects the short-term response of photosynthesis to fluctuating irradiance. This definition is in agreement with recent literature on acclimation to irradiance (Athanasiou et al., 2010; Dietz, 2015; Vialet-Chabrand et al., 2017; Yin et al., 2019), though we acknowledge that some studies on acclimation may only focus on reversible physiological processes (Walters, 2005; Caliandro et al., 2013; Miller et al., 2017; Albanese et al., 2018).

### Types of Acclimation

The short-term response of leaf photosynthesis varies over time, both diurnally and seasonally (Figure 1). Diurnal changes are driven by changes in environmental factors and circadian rhythms (Resco de Dios and Gessler, 2018). There are two types of acclimatory processes in leaves: (i) developmental acclimation during leaf development, which determines anatomical and biochemical traits and (ii) dynamic (physiological) acclimation after the leaf is fully expanded, whereby leaf N in pigments and proteins and biochemical composition of the chloroplasts (i.e., the relative amounts of pigments and proteins involved in photosynthesis) may change further (Athanasiou et al., 2010). In *Chenopodium album*, developmental acclimation of a growing leaf responded to the irradiance incident on mature, fully expanded leaves (Yano and Terashima, 2001). The same phenomenon was observed in *Glycine max* (Wu et al., 2018), *Phaseolus vulgaris* (Murakami et al., 2014), *Helianthus annuus* (Yamazaki and Shinomiya, 2013), *Sorghum bicolor* (Jiang et al., 2011), and Arabidopsis (Munekage et al., 2015). When mature leaves where shaded and growing leaves exposed to high irradiance, the leaf traits (i.e., leaf thickness, stomatal density, total N) reflected the irradiance level on mature leaves, whereas biochemical composition and chloroplast ultrastructure responded to irradiance absorbed by the growing leaf (Yano and Terashima, 2001; Jiang et al., 2011; Yamazaki and Shinomiya, 2013). This suggests different mechanisms, whereby developmental acclimation may be regulated by long distance signals (Munekage et al., 2015), while dynamic acclimation and changes in chloroplast composition are controlled locally, most likely by retrograde signaling from the chloroplast.

### Chloroplast Retrograde Signaling and Gene Expression

Periods of high irradiance enable higher rates of electron and proton transport and CO_2_ fixation, but also cause photooxidative stress. Multiple signaling components arising in the chloroplast, such as the plastoquinone redox state, photosynthetic metabolites, reactive oxygen species (ROS), sugars, and hormones, act on multiple time scales in pathways that trigger changes in chloroplast (Mullet, 1993; Pfannschmidt et al., 1999) and in nuclear gene expression, the latter through chloroplast to nucleus (i.e., retrograde) signaling. These changes in expression lead to subsequent changes in protein abundance that are associated with dynamic acclimation to fluctuating irradiance (Dietz, 2015; Chan et al., 2016). In the first seconds after an increase in irradiance, faster linear electron transport increases the concentrations of plastoquinol and reduced thioredoxin, Calvin Bassham Benson (CBB) cycle metabolites (Vogel et al., 2014) and glutathione (Choudhury et al., 2018b), which may participate in retrograde signaling. During these first seconds, singlet oxygen (^1^O_2_) is produced in the photosystem II reaction center and, although it is unlikely to diffuse out of the cytosol, it can reduce β-carotene to β-cyclocitral, which may trigger changes in nuclear gene expression (Matsubara et al., 2016). Within minutes, hydrogen peroxide (H_2_O_2_) levels increase due to the activity of superoxide dismutase (Mubarakshina et al., 2010; Choudhury et al., 2018a); H_2_O_2_ can then diffuse into the cytosol and interact with several nuclear gene expression mediators (Pfalz et al., 2012). Also, in the minute to hour domain, levels of the phytohormones abscisic acid (ABA; Galvez-Valdivieso et al., 2009), jasmonic acid, and its precursor oxophytodienoic acid, as well as that of methylerythritol cyclodiphosphate, rise (Alsharafa et al., 2014). Sugars, salicylic acid, auxin, and gibberellic acid are to respond last, with their concentrations rising hours after an irradiance was increased. For more comprehensive reviews, the reader is referred to Dietz (2015) and Chan et al. (2016).

### Systemic Acquired Acclimation

Upon abiotic stress (including high irradiance), signals not only flow from chloroplasts to the nucleus inside the same cell, but also from exposed (target) leaves to non-exposed (systemic) plant organs, in a process termed SAA (Mittler and Blumwald, 2015). Signals triggering SAA include ROS waves (Gechev et al., 2006), calcium waves, hydraulic waves, electric signals, and ABA (Mittler and Blumwald, 2015), and the calcium, ROS, and electric wave are likely linked to propagate and reinforce one another (Gilroy et al., 2016). A large range of metabolites increased in systemic tissues within 1–12 min of high irradiance (1500 μmol m^–2^ s^–1^) exposure of a target leaf in Arabidopsis (Choudhury et al., 2018a), triggering changes in several thousand gene transcripts in the systemic leaf within minutes (Zandalinas et al., 2019). Systemic signals such as H_2_O_2_, either directly applied or triggered through high light stress, have been shown to make target leaves more resistant to subsequent stress (Karpinski et al., 1999), including pathogen attacks (Karpinski et al., 2013). Clearly, both local and global signaling and gene expression respond rapidly and massively to high irradiance stress, to prepare the plant for future stresses.

### Light Signaling Under Fluctuating Irradiance: A Role for Photoreceptors?

Photoreceptors such as phytochromes could be another signaling system for sunflecks. For example, phytochrome B is known to sense neighboring plants through the red:far red ratio, and to trigger subsequent shade avoidance responses which strongly impact on plant morphology (Ballaré and Pierik, 2017). Exposure to FR typically increases whole-plant irradiance capture (increased stem and leaf elongation), tends to decrease leaf photosynthetic capacity (e.g., Ji et al., 2019), and may affect the distribution of canopy-wide irradiance fluctuations.

Rapid transitions between shade and full sunlight in the field do not only change the irradiance a plant is exposed to, but also R/FR, thereby impinging on the phytochrome photostationary state. Indeed, 2 h high-irradiance periods in the field, during which irradiance increased 10- to 30-fold and R/FR increased 10-fold, reduced the shade avoidance reactions (hypocotyl elongation) in Arabidopsis WT, but not in *phyAphyB* double mutants (Sellaro et al., 2011). A subsequent study (Sellaro et al., 2019) modeled the kinetics of phytochrome B conversion between its active and inactive forms, and predicted responses in hypocotyl growth to R/FR experiments, suggesting that the concentrations of active and inactive forms of phytochrome B are affected by fluctuations in irradiance. Interestingly, their experimental data (Figure 3A in Sellaro et al., 2019) suggested that nuclear phytochrome B abundance increases with irradiance. Additionally, Franklin et al. (2007) showed synergistic regulation of hypocotyl elongation in response to different red irradiances by phytochromes A and B, suggesting that these photoreceptors do not only respond to changes in light quality, but also quantity. These results hint that phytochromes do not only act as sensors of light spectrum and temperature (Jung et al., 2016; Legris et al., 2016), but that they may additionally be responsive to changes in irradiance alone.

In addition to shade avoidance responses, high R/FR will result in an imbalance between the two photosystems responsible for light capture in photosynthesis due to their different spectra of absorbance. In the short term, this imbalance can be compensated for by state transitions that will transfer pigments between photosystems but in the long term it will result in acclimation of the photosynthetic apparatus through changes in the stoichiometry of protein complexes and pigments (Walters and Horton, 1995; Dietzel et al., 2008). However, this experimental evidence was acquired under constant irradiance conditions during daytime and, to our knowledge, the effect of R/FR on photosynthetic acclimation under fluctuating irradiance has not been studied yet.

### Gene Expression

Nuclear gene expression reacts to irradiance in a highly dynamic way: hundreds of transcripts change within seconds-minutes after increases (Suzuki et al., 2015) and decreases (Crisp et al., 2017) in irradiance, suggesting that under a naturally fluctuating irradiance, gene expression will also be strongly affected. Indeed, a recent ground-breaking study (Schneider et al., 2019) has demonstrated the impact of fluctuating irradiance on gene expression in Arabidopsis. Short and strong light pulses, applied repeatedly for 3 d, caused a differential expression (DE) of ∼4000 genes, 75% of which were upregulated. Chloroplast components were mostly found among upregulated genes whereas genes encoding for ribosomes, the Golgi apparatus, and cell wall components were more strongly downregulated. Half of the genes that were upregulated in young leaves were also upregulated in leaves inoculated with *Pseudomonas syringae*, suggesting that exposure to fluctuating irradiance may prime plants for biotic stress. Large effects of time of day and leaf developmental stage were observed: for example, many genes encoding for light harvesting complex proteins were downregulated in young leaves in the evening whereas a large number of genes involved in photosynthesis, photoprotection, and photorespiration were specifically upregulated in old leaves, but only in the evening. Gene expression seems to be coordinated by circadian rhythms, as explained above.

Of all 4000 DE genes, only 46 were shared between all samples, i.e. in young and mature leaves and at both times of sampling (morning and evening). These central genes included genes for components of light harvesting (LHCB7), CBB enzymes (sedoheptulose-1,7-bisphosphatase, fructose-1,6-bisphosphate, and CP12), the photorespiratory pathway and ROS metabolism (glycolate oxidase, catalase), CO_2_ interconversion (beta carbonic anhydrase), sucrose transport (sucrose-phosphate synthase C), photooxidative stress responses (activity of BC1 complex kinases, fatty acid desaturases, vitamin 6 biosynthesis, and glutathione peroxidase), and photoreceptor interacting factors involved in photomorphogenesis (blue light inhibitor of cryptochromes 1, HY5-homolog, B-box domain 17 protein).

The molecular response to fluctuating irradiance goes far beyond that of photooxidative stress: the data by Schneider et al. (2019) suggested that processes regulating plant growth are affected on many levels, and that the expression of the respective genes is further under strong circadian and developmental control. However, altered gene expression does not necessarily equate altered protein abundance. Also, changes in protein contents, enabling effective acclimation, may take several more days to take effect (Athanasiou et al., 2010).

### Protein Abundance and Canopy-Wide N Distribution

Information on changes in protein abundance due to acclimation to fluctuating irradiance is scarce. For Arabidopsis exposed to lightflecks for 7 d, Caliandro et al. (2013) found that chlorophylls decreased, carotenoids remained unchanged, and the PsbS protein (involved in non-photochemical quenching) increased. However, since Caliandro et al. (2013) only looked at these proteins, it is not clear whether other proteins may be affected by irradiance fluctuations, too. Some ideas may be derived from proteomics studies of high light acclimation (Miller et al., 2017) 100–400 μmol m^–2^ s^–1^ for 7 d, Arabidopsis) and growth of *Pisum sativum* at 30, 150, and 750 μmol m^–2^ s^–1^ (Albanese et al., 2018). In Miller et al. (2017), dynamic high irradiance acclimation entailed a strong increase in most proteins (1284 out of 1993 proteins increased, 14 decreased). In the chloroplast electron transport chain, high irradiance caused a reorganization (but not an increase) in both photosystems, as well as increases in the abundance of cytochrome b_6_f complex proteins, plastocyanin, the ferredoxin NADP^+^ reductase, and several ATP synthase subunits (Miller et al., 2017; Albanese et al., 2018). Further, there were increases in PsbS and the violaxanthin de-epoxidase (Miller et al., 2017). Downstream of the electron transport chain, CBB enzymes were increased in abundance (on average by 50%), as were nearly all enzymes belonging to starch and sucrose metabolism (Miller et al., 2017).

After a leaf is fully expanded, further dynamic acclimation may occur, but this is constrained by leaf anatomy (Oguchi et al., 2005) and N distribution in the canopy (Kull, 2002). Redistribution of N within canopies results in vertical profiles of total leaf N and photosynthetic capacity that theoretically should approximate average irradiance profiles (Hikosaka et al., 2016), as this would maximize canopy photosynthesis for a given set of environmental conditions and total canopy N content (Field, 1983; Farquhar, 1989). However, canopies often display shallower profiles, indicating supraoptimal amounts of N and photosynthetic capacity in the lower leaves of a canopy (Anten, 2016; Hikosaka et al., 2016). This behavior can be explained in evolutionary terms, either because the species being analyzed evolved in a different environment (e.g., crops in intensive agriculture) or because the fitness functions driving natural selection are more complex than the instantaneous rate of canopy photosynthesis (Anten, 2016). Nevertheless, considering irradiance fluctuations provides novel insights into the analysis of acclimation at the canopy level with the use of optimization algorithms.

Retkute et al. (2015) suggested that the leaf optimal photosynthetic capacity under fluctuating irradiance depends on the frequency and amplitude of fluctuations for the same average irradiance, resulting in sub-optimal photosynthetic acclimation in wheat canopies (Townsend et al., 2018). Mott and Woodrow (2000) explored the optimal partitioning of N between Rubisco and Rubisco activase, suggesting that the optimal partitioning between the two is highly dependent on the duration of the fluctuations (shorter durations meaning a higher ratio of Rubisco activase to Rubisco).

A general issue with these model-based analyses is that they tend to oversimplify the dynamic responses of photosynthesis by using a single rate constant, thereby assuming a single limiting mechanism. However, the different mechanisms limiting dynamic responses of photosynthesis are characterized by different rate constants and their relative importance depends on the frequency of fluctuations (Morales et al., 2018a). Although detailed dynamic models of C3 photosynthesis exist (Zhu et al., 2013; Morales et al., 2018a, b), no comprehensive optimization analysis of photosynthesis under fluctuating irradiance has been published thus far.

### Genetic Diversity of Acclimation

There is genetic diversity in dynamic acclimation to a change in irradiance (Athanasiou et al., 2010; Rooijen et al., 2015), but this remains unexplored with regards to fluctuating irradiance. The ability to undergo dynamic acclimation in Arabidopsis has been linked to the glucose-6-phosphate/phosphate translocator across the chloroplast envelope (Athanasiou et al., 2010; Dyson et al., 2015; Miller et al., 2017) and loss-of-function mutants in this gene had significantly lower fitness when grown under fluctuating irradiance (Athanasiou et al., 2010). The increase in activity of this transporter in response to an increase in irradiance could result in the import of glucose-6-phosphate into the chloroplast, which would stabilize photosynthetic metabolism during acclimation (Weise et al., 2019) but it is still unclear why its expression is required for dynamic acclimation to occur.

### Dynamic Acclimation May Never Reach a Steady State

Dynamic acclimation in response to a change in irradiance can take days to take place (Athanasiou et al., 2010; Rooijen et al., 2015). Since fluctuations in irradiance are faster, even at the seasonal level, it is possible that plants never reach full acclimation and remain in an intermediate, dynamic equilibrium state. This equilibrium state would depend on the speed of dynamic acclimation and the degree of linearity in the response of plant traits to changes in irradiance. These dynamics have been captured in simulation models either (i) by implementing a goal-seeking behavior that calculates steady-state acclimation from optimization theory (Yin et al., 2019) or (ii) by simulating protein turnover dynamically (Thornley, 1998; Kull and Kruijt, 1999; Barillot et al., 2016; Pao et al., 2019a). Experimental evidence exists that coordination across leaves may be achieved through cytokinins carried by the transpiration stream (Pons et al., 2001), as transpiration will vary according to the irradiance profile. This mechanism has been included in a recent mechanistic model of wheat incorporating plant carbon and N balances, but has not yet been validated experimentally (Barillot et al., 2016).

## Experimentation on Acclimation to Fluctuating Irradiance: Methodology and Summarized Results

### Methodology

In several groundbreaking pioneer studies, relatively simple experiments were used to test the effects of various molecular players on plant growth and fitness under fluctuating irradiance: Mutants lacking components of energy quenching (*npq1*, *npq4*) and state transitions (*stn7*) showed reductions in fitness (i.e., number of seeds produced) and/or biomass relative to the wild-type, when grown under fluctuating light (Kühlheim et al., 2002; Bellafiore et al., 2005; Kühlheim and Jansson, 2005; Frenkel et al., 2007; Wagner et al., 2008). Fluctuating light was supplied naturally, in the field (Kühlheim et al., 2002; Kühlheim and Jansson, 2005; Frenkel et al., 2007) and/or in controlled climate chamber experiments (Kühlheim et al., 2002; Bellafiore et al., 2005; Wagner et al., 2008). In none of these cases was a control treatment with constant light used in which the average intensity and spectrum were identical to that of the fluctuating light treatment; given that these experiments were aimed at characterizing the (relatively strong) effects of specific and well-characterized mutations on FL acclimation, this approach can be justified. However, if an experiment is to accurately quantify the (sometimes small) effects of irradiance fluctuations on wild-type plants, it requires a control where irradiance is constant throughout the photoperiod, and whose average intensity and spectrum are the same as that of the treatment(s) containing irradiance fluctuations. Also, the experimenter needs to be in full control of intensity, timing, and frequency of the irradiance fluctuations. From these requirements it follows that such experiments must be done under controlled growth conditions and in the absence of natural background irradiance (i.e., not in the field or greenhouse). Several such experiments have been performed (Watling et al., 1997; Leakey et al., 2002; Kubásek et al., 2013; Cruz et al., 2016; Annunziata et al., 2017, 2018; Vialet-Chabrand et al., 2017; Kaiser et al., 2018a; Matthews et al., 2018) and some of their results are analyzed below.

Most fluctuating irradiance regimes have been achieved by modulating intensity of an artificial light source in plant growth chambers or cabinets (Watling et al., 1997; Kubásek et al., 2013; Cruz et al., 2016; Annunziata et al., 2017, 2018; Vialet-Chabrand et al., 2017; Matthews et al., 2018). However, other methods have been employed, including moving light sources over the plants (Zheng et al., 2006; Blom and Zheng, 2009; Kaiser et al., 2018a) and rotating shading discs that transiently block the light sources (Leakey et al., 2002). Shading discs are perhaps logistically more complex to implement, but they can be used to alter the spectrum of the artificial light at the same time as the irradiance (although, to our knowledge, they have not been used with that purpose). The use of moving light sources differs from the rest, as it introduces changes in the angle of incidence of the irradiance in addition to fluctuations in the irradiance level.

The fluctuations in irradiance employed in these experiments can be classified into two categories: (i) experiments that focused on diurnal variation of irradiance and (ii) experiments that focused on rapid, repeated fluctuations (denoted as lightflecks, to distinguish between the natural fluctuations such as sunflecks and cloudflecks). The diurnal variation of irradiance has been approximated with a sinusoidal pattern (half the period of a sine wave) where the maximum occurs in the middle of the photoperiod (Cruz et al., 2016; Annunziata et al., 2017, 2018; Matthews et al., 2018). Rapid fluctuations were most often implemented by adding periods of high irradiance on top of a constant, low irradiance, background (Watling et al., 1997; Leakey et al., 2002) or on top of a sinuosidal pattern (Cruz et al., 2016; Matthews et al., 2018). Exceptions include experiments based on moving light sources (Kaiser et al., 2018a) and experiments that mimic time series of irradiance measured outdoors (Vialet-Chabrand et al., 2017; Matthews et al., 2018).

For studies that focused on rapid fluctuations, the shortest duration for a lightfleck was 20 s (Kaiser et al., 2018a) while most studies used lightflecks of ≥180 s. As discussed above, a typical duration for a sunfleck is <2 s (Kaiser et al., 2018b), meaning that these experiments have not used the correct timescale if the objective was to study the response of plants to the most frequently occurring sunflecks. Also, lightflecks in these experiments resemble cloudflecks rather than sunflecks, in the sense that the fluctuations are applied to the light source itself, rather than as a result of change in incident angle, gaps in the canopy or wind-induced plant movements.

### Summary of Observed Responses to Fluctuating Irradiance

To explore whether plant traits at the various integration levels respond in a similar manner across species, we compiled published data on some of the most frequently measured traits, i.e. the chlorophyll a/b ratio (chloroplast level), specific leaf area (SLA, cm^2^ g^–1^), light saturated net photosynthesis rate (*A*_*max*_; leaf level), root/shoot ratio, and plant biomass. For this analysis, we focused on studies with (near-) identical average irradiance and spectrum between treatments, such that the effect may be caused by irradiance pattern alone, i.e., constant (C) vs. fluctuating irradiance (F). Using this criterium allowed us to include 43 data sets from six studies (Supplementary Table S1; Watling et al., 1997; Leakey et al., 2002; Grieco et al., 2012; Kubásek et al., 2013; Vialet-Chabrand et al., 2017; Kaiser et al., 2018a). Unfortunately, many studies were excluded from this analysis as they either (i) did not contain a constant irradiance treatment, (ii) did not ensure that average irradiance between treatments was identical, or (iii) measured traits that were not reported in a sufficient number of other studies to allow for a cross-study comparison. Data were analyzed correcting for variability of individual data sets and number of biological replicates, and relative effects of fluctuating light were then expressed as (*F*−*C*)/*C*, where *F* and *C* denote the average trait value under fluctuating and constant irradiance, respectively.

**FIGURE 2:**
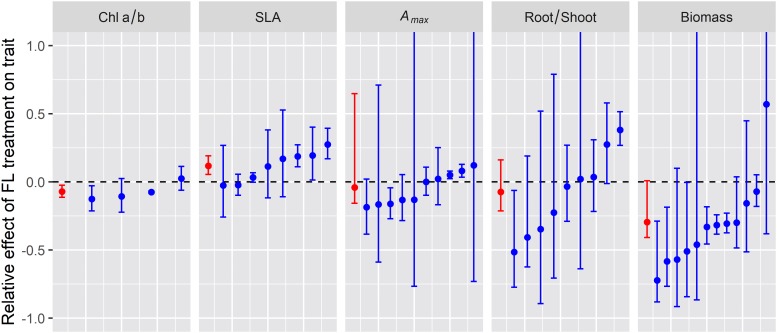
Relative effect of fluctuating irradiance (FL) treatment on plan traits for the different experiments reviewed (blue symbols and error bars) and the average relative effect across experiments (red symbol and error bars). Error bars indicate 95% confidence intervals (i.e., the 2.5 and 97.5% of the distribution of relative effect) whereas symbols indicate the median relative effect.

Chlorophyll a/b ratio was significantly lower, on average by 7%, in leaves grown under lightflecks (Figure 2; *n* = 4). These results suggest a relative increase of light harvesting (associated with Chl b) over reaction center complexes in photosystem II (associated with Chl a) in leaves under fluctuating irradiance, which is typically observed in shade-acclimated leaves (Schöttler and Tóth, 2014; Albanese et al., 2018). However, our numbers are based on only four studies, three of which were conducted on Arabidopsis. The reduction in Chl a/b ratio may be species specific. Indeed, in the study using *Shorea leprosula* instead of Arabidopsis, Chl a/b was unaffected (Leakey et al., 2002).

Specific leaf area was 12% higher in lightfleck acclimated leaves (Figure 2, *n* = 8), implying that the formation of leaves with a reduced biomass per area under fluctuating irradiance was a generic response, which again is reminiscent of shade acclimation (Evans and Poorter, 2001).

Photosynthetic capacity, here expressed as light-saturated CO_2_ assimilation (*A*_*max*_), was not generally affected by fluctuating irradiance (Figure 2, *n* = 10). These lightfleck effects may be species-specific: when grown under identical treatments, *A*_*max*_ in petunia (*Petunia* × *hybrida*) and tomato (*Solanum lycopersicum*) increased significantly under fluctuating irradiance, while in chrysanthemum (*Chrysanthemum morifolium*) it did not (Blom and Zheng, 2009). Given the large differences between species in high irradiance acclimation capacity (Murchie and Horton, 1997), this result may be unsurprising. Nevertheless, these data are in contrast to a modeling study by Retkute et al. (2015) which indicated that the optimal plant response to fluctuating irradiance was to increase *A*_*max*_. These results may also be in disagreement with a recent commentary by Pao et al. (2019b), who suggested that photosynthetic capacity (expressed as maximum electron transport and carboxylation rates, *J*_*max*_ and *V*_*cmax*_, respectively) is reduced in leaves under fluctuating irradiance; this analysis was based on a non-linear relationship between irradiance and protein synthesis rate, using data from a previous modeling study validated with measurements on *Cucumis sativa* (grown under constant irradiance) as reference (Pao et al., 2019a). The reasoning by Pao et al. (2019b) is that leaves under fluctuating irradiance experience a relatively longer time close to the saturating end of this relationship compared to leaves under lower, uniform light. We did not find a sufficient number of datasets on *J*_*max*_ or *V*_*cmax*_ in studies on lightfleck acclimation to draw robust conclusions, but assuming that a reduction in *J*_*max*_ and *V*_*cmax*_ would coincide with a reduction *A*_*max*_, we can at least conclude that it is unlikely that *J*_*max*_ and *V*_*cmax*_ will generally decrease under fluctuating irradiance.

There was a tendency for a decrease in the root/shoot ratio under fluctuating irradiance, but this was not significant (Figure 2, *n* = 9). Similarly to *A*_*max*_, this trait seemed to be under strong genetic control, as under identical treatments wheat (*Triticum aestivum*), *Setaria macrostachya*, and *Amaranthus caudatus* showed strong (37–52%) decreases in the root/shoot ratio under fluctuating irradiance, whereas *Celosia argentea* showed no response (Kubásek et al., 2013).

Plant biomass was significantly reduced under fluctuating irradiance, by 32%, although there was large variability around the mean (Figure 2; *n* = 12). At first glance, this reduction may seem obvious, given that (a) photosynthesis reacts non-instantaneously to an increase in irradiance due to photosynthetic induction and thereby has a lower time-integrated CO_2_ assimilation compared to the steady state (Kaiser et al., 2015, 2018b) and (b) due to the saturating, non-linear response of steady-state leaf photosynthesis to irradiance, fluctuating irradiance treatments typically expose the leaf to a larger fraction of high irradiance that is used with a lower quantum efficiency compared to the uniform, low irradiance controls. However, it is noteworthy that as with *A*_*max*_ and the root/shoot ratio, different species in the same experiment displayed very different plant biomass responses to fluctuating irradiance: while biomass was significantly reduced (−21%) in tomato under fluctuating irradiance, in chrysanthemum, petunia and rose (*Rosa* × *hybrida*) it was unaffected (Blom and Zheng, 2009). These results hint at the possibility that acclimation to fluctuating irradiance may counteract the negative effects of fluctuating irradiance on biomass, and that the capacity for this compensatory acclimation may be species dependent and could therefore be used as a breeding target.

Some of the reported effects of fluctuating light on treatments coincide with the effects expected from a low irradiance treatment (i.e., acclimation to low irradiance) including higher SLA and lower chlorophyll a/b ratio (Figure 2), but *A*_*max*_ did not change or even increased (unlike acclimation to low irradiance when it would always decrease). Therefore, acclimation to fluctuating light seems to differ from acclimation to low irradiance although some traits may respond similarly.

The experiments reviewed maintained the same average irradiance in the constant and fluctuating light treatments. This means that plants under fluctuating light were being exposed to lower irradiance than in the control during part of the daytime. However, the fraction of the daytime when this was the case varied across studies (Supplementary Table S1) from approximately 50% (Vialet-Chabrand et al., 2017) to >83% (Watling et al., 1997; Kaiser et al., 2018a). There was a weak negative correlation (−0.33) between *A*_*max*_ and the fraction of the daytime where irradiance was lower (Supplementary Table S1), but stronger correlations for chlorophyll a/b ratio and SLA (0.64 and −0.65, respectively). This means that, across the different experiments, a longer exposure to lower irradiance resulted in a smaller effect of fluctuating irradiance on chlorophyll a/b ratio and SLA but a stronger effect on *A*_*max*_. This trend further reinforces the hypothesis that the changes observed in the different traits are not due to acclimation to the low irradiance periods of the fluctuating light treatment.

Although these data suggest that acclimation to fluctuating irradiance is distinct from acclimation to low irradiance, the experiments reviewed used different plant species, average irradiance levels, and dynamic patterns of oscillations, so confounding effects cannot be discarded. We suggest that future experiments on fluctuating irradiance include (when relevant) a second control where plants are grown at a constant, low irradiance level equal to the prevailing background irradiance of the fluctuating treatment, to further clarify the role of low irradiance in acclimation to fluctuating irradiance.

## Outlook

The data summarized here suggest that acclimation to fluctuating irradiance resembles that of shade acclimation for some traits. However, we are still lacking (insights from) studies that expose plants to several combinations of lightfleck timing, frequency, amplitude, and absolute intensity, to fully understand what the drivers for acclimation of a given trait are. Such studies should also account for the genetic variation that exists for the capacity to change a given trait during acclimation. Further, we emphasize once more the need for experimental setups that ensure that the average irradiance (and spectrum) between treatments is identical and that a treatment with constant irradiance is included. Recent advances in LED technology (Pattison et al., 2018) that allow for accurate and rapid modulation of intensity, as well as for emulating the natural light spectra, are instrumental to advancement of this field of research. Finally, many studies, e.g., those cited on retrograde signaling, SAA, and gene transcription were conducted under relatively extreme conditions: plants were grown under a very low irradiance (2–5% of full sunlight) and then were exposed to 50–100% of full sunlight to trigger a change. The molecular responses in these studies were indeed intriguingly rapid and massive, but these may be weaker in field-grown plants that are acclimated to a stronger, and more fluctuating, irradiance.

## Author Contributions

All authors listed have made a substantial, direct and intellectual contribution to the work, and approved it for publication.

## Conflict of Interest

The authors declare that the research was conducted in the absence of any commercial or financial relationships that could be construed as a potential conflict of interest.
